# Microwave-assisted Synthesis of Hexagonal Gold Nanoparticles Reduced by Organosilane (3-Mercaptopropyl)trimethoxysilane

**DOI:** 10.3390/ma12101680

**Published:** 2019-05-23

**Authors:** Kwok Wei Shah, Long Zheng

**Affiliations:** School of Design and Environment, National University of Singapore, 4 Architecture Drive, Singapore 117566, Singapore

**Keywords:** gold nanoparticles, nanoparticles, colloids, nanosynthesis, chemical reduction microwave synthesis

## Abstract

A novel synthesis of hexagonal gold nanoparticles (Au NPs) via hydrolyzed organosilane (3-mercaptopropyl)trimethoxysilane (MPTMS) using an ultrafast and environmentally friendly method is presented in this study. For the first time, organosilane MPTMS is used for chemical reduction of auric acid under ultrafast microwave irradiation. To the best of our knowledge, the use of organosilane for the synthesis of Au NPs has not been reported. The entire one-step process is convenient, rapid and cost-effective, as well as eco-friendly under alcohol-free aqueous media. Different characterization methods were carried out to investigate the properties of synthesized gold nanoparticles. transmission electron microscopy and scanning electron microscopy were used to investigate the morphology of as-synthesized Au NPs, while X-ray powder diffraction was applied to obtain the crystalline nature. Nuclear magnetic resonance was used to track the hydrolysis of organosilane MPTMS, which is employed for the first time as a reducing agent for the synthesis of Au NPs. The impact from microwave irradiation time and power, as well as the catalytic property of as-synthesized Au NPs, was investigated via ultraviolet–visible spectroscopy. The as-synthesized products include gold nanohexagon and two-dimensional hexagonal gold nanoplatelets, both of which are single-crystal with (1 1 1) planes as basal surfaces. From UV-vis spectra, it is found that the facile water-based fabrication of hexagonal Au NPs began within seconds of microwave irradiation and the size growth increased with the microwave power and time. Moreover, the efficient reduction of 4-nitrophenol to 4-aminophenol in the presence of as-synthesized Au NPs was observed, exhibiting a remarkable catalytic activity. The present simple, rapid and convenient one-step microwave process possess high scalability and useful for future applications such as catalysis, medical, biological, plasmonic sensors and electronics.

## 1. Introduction

Gold nanoparticles (Au NPs) are versatile metallic nanostructures with excellent electronic, physical, chemical and optical properties due to well-developed synthetic procedures [1,2,3]. Their unique properties have been utilized in a broad range of high technology applications such as catalysis [4,5,6], sensor probes [7,8], drug delivery [9], therapeutic agents [10,11,12], electronic conductors [9], organic photovoltaics [13,14,15] and glass coatings [16,17,18]. These versatile applications of Au NPs are mainly related to the various morphologies of Au NPs, because different morphologies usually lead to very different properties, such as surface atom densities, electronic structures and probable chemical reactivities. Currently, there are many reports on the synthesis of Au NPs by other research groups. For example, Wei et al. [19] have utilized chitosan and successfully synthesized single-crystal gold nanosheets while Yang et al. [20] have readily prepared gold nanostructures (tetrahedra, cubes, octahedra, and icosahedra) via the polyvinylpyrrolidone (PVP) mediated polyol process with high yield and good uniformity, and Tsuji et al. [21] have obtained polygonal gold nanoplates, rods, and wires via a polyol process. However, these methods can be slow, expensive and tedious, because current procedure involves many time-consuming and highly costed steps, while the reducing agents and solvents used are somehow toxic and/or harmful to the environment during the preparation, not satisfied for a green synthesis process [22,23,24,25]. Traditional synthesis of Au NPs is usually by reduction of gold salts in thermal aqueous solutions which is heated in oil bath and refluxed for many hours and the most conventional reducing agents are sodium citrate or sodium borohydride, etc [26,27,28,29]. The resulting final products are always a mixture of Au NPs in various sizes and shapes [21,30,31,32,33]. 

Herein, a novel water-based synthesis strategy using organosilane (3-mercaptopropyl)trimethoxysilane as reducing agent and microwave irradiation as fast heating source to produce hexagonal Au NPs in an economically effective and energy-efficient manner is reported. To the best of our knowledge, the use of organosilane for the synthesis of Au NPs has not been reported. Additionally, microwave heating is applied to not only reduce reaction time dramatically, but also avoid side reaction due to selective heating [32,34,35,36,37]. Compared to conventional convective heating method from external heat source to the reactants ultimately and with large thermal gradient and poor nucleation, microwave method provides selective heating, localized rapid and dielectric heating internally and directly target nucleates to maintain uniform nucleation and growth rates for better product quality [38,39]. The combined use of organosilane as the reducing agent and microwave as fast heating source could achieve cost- and energy-effective synthesis in one-pot process. Unlike other alcohol-based synthetic approach involving the use of harmful alcoholic solvent and toxic chemicals [40,41], this fully water-based “green synthesis” route could fill the growth need of environmental friendly experiment procedure.

## 2. Experimental Section

### 2.1. Materials

(3-Mercaptopropyl)trimethoxysilane (MPTMS, >99.5%), gold chloride trihydrate (HAuCl_4_∙3H_2_O), sodium borohydride (NaBH_4_, >99%), and 4-Nitrophenol (>99%) were purchased from Sigma-Aldrich. All chemicals were at reagent grade and used without further purification. All aqueous solutions were prepared using deionized water. 

### 2.2. Synthesis 

Au NPs were prepared using a domestic microwave (MW) oven (Sharp R202ZS, maximum power of 800W, Osaka, Japan). In a typical synthesis, 100µl MPTMS was added to 10 ml DI water and stirred over night to obtain hydrolyzed MPTMS solution (stock solution). 40 µl of such stock solution and 1mg HAuCl_4_ were then added to 3 ml of deionized (DI) water. The mixture was under MW irradiation until red-purple color was observed. Typically under 800W MW irradiation power, the mixture of 40 µl stock solution and 1 mg HAuCl_4_ in 3 ml DI water could complete the reaction within 60 seconds to derive hexagonal Au NPs.

In order to investigate the impact of irradiation time on Au NP synthesis, six samples were prepared under MW irradiation for different time periods. Specifically, the mixture of 240 µl stock solution and 6mg HAuCl_4_ in 18 ml DI water was under MW irradiation from 0 second to 60 second with interval of 10 seconds. Every 10 seconds, 3 ml of the mixture was taken out and stored in glass vial. To study the impact of MW power, four samples were prepared under MW irradiation with various power. Four identical mixtures of 40 µl stock solution (2.15 µmol) and 1mg HAuCl_4_ (2.95 µmol) in 3 ml DI water were under MW irradiation for 20 seconds with the power of 200 W, 400 W, 600 W and 800 W, respectively. After MW irradiation, the as-obtained NPs were centrifuged at 4000 rpm and washed with DI water three times. The purified Au NPs were dispersed in water for further characterization.

The catalytic efficacy of as-synthesized Au NPs were further investigated. In a typical reduction reaction of 4-nitrophenol (4-NP), 1 ml of as-synthesized Au NP solution was mixed with 1 ml of 0.04 M NaBH_4_, followed by adding 0.3 ml of 2 mM solution of 4-NP. At every 5 minutes’ interval, the reduction progress of 4-NP was monitored via UV-vis spectroscopy.

### 2.3. Characterization

The morphology of Au NPs was investigated by transmission electron microscopy (TEM) machine (JEOL, JEM 2010F, operating voltage of 200kV, Tokyo, Japan) and scanning electron microscope (SEM) machine (Hitachi, SU8020, operating voltage of 20kV). The crystal nature of AuNPs was tested using X-ray diffraction (XRD) with X-ray Powder Diffraction a D/max-2400 diffractometer (Rigaku, Tokyo, Japan) in the diffraction angle range of 30–90°. The UV–vis spectra were recorded through Shimadzu UV-3150, UV–vis spectrophotometer. ^29^Si nuclear magnetic resonance (NMR) spectra of MPTMS monomer and hydrolyzed MPTMS monomer were recorded by 400-MHz Advance III (4.1 T) spectrometer (Bruker, Billerica, MA, USA). 

## 3. Results and discussion

### 3.1. Formation of Au NPs

**Figure 1:**
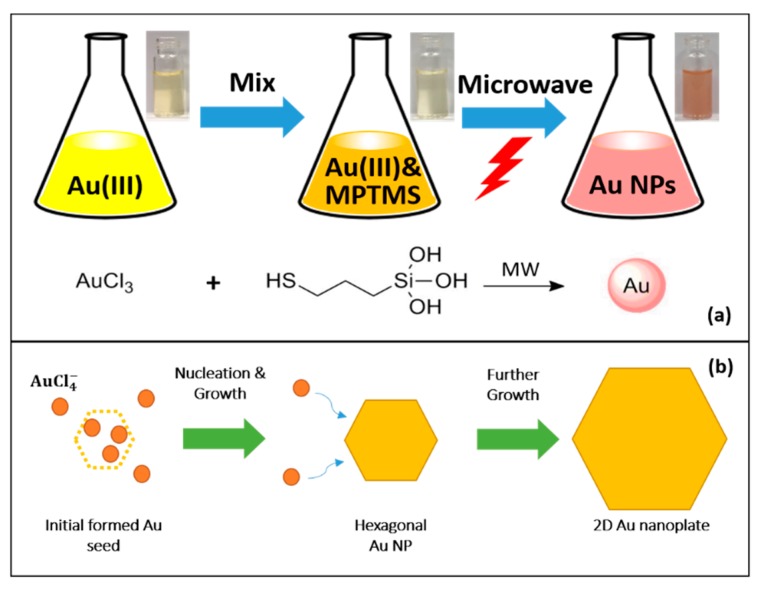
(**a**) Chemical procedure for synthesis of hexagonal gold nanoparticles (Au NPs). (**b**) Schematics of Au NPs growth and formation.

For a typical synthesis procedure, hydrolyzed MPTMS was prepared by mixing MPTMS and DI water and stirring overnight before the experiment. Subsequently, a simple one-step synthesis (Figure 1) was used to derive hexagonal Au NPs. HAuCl_4_ and hydrolyzed MPTMS were firstly mixed in aqueous solution, followed by the irradiation under MW. The color of the mixture solution was changed gradually after approximate 10–20 seconds’ MW irradiation, indicating the formation of Au NPs immediately. 

In this reaction, it is believed that hydrolyzed MPTMS acts as a reduction agent in the reaction for Au NPs synthesis. AuCl_4_^−^ ions are reduced to Au^0^ under MW irradiation by hydrolyzed MPTMS, because MPTMS is hydrolyzed and thus equipped with reducing property due to the high reactivity of silane. More specifically, Au monomers are first obtained by reducing AuCl_4_^−^ ions under MW irradiation during the reaction. Then, Au starts to nucleate and grow into particles with controllable morphology quickly when the concentration of Au atoms reaches supersaturation. In this process, hydrolysis of MPTMS silanes forms silanol groups, followed by free radical reduction to produce Au NP nuclei. The formation of Au NPs is proposed to take place by the reaction of auric acid with reductive free radical agents including hydroxyl (OH^−^) ions and hydrogen (H^+^) ions released by hydrolysis of MPTMS. This is consistent to other similar reported works on the reducing properties of reactive silanes [42,43,44,45]. In the study from Motoyama et al. [42], different silanes, including poly-(methylhydrosiloxane) (PMHS) and 1,1,3,3-tetramethyldisiloxane (TMDS) were used with iridium-based catalyst by to reduce carboxamide, while octadecylsilane (ODS) was used by Chauhan et al. [43] as the reducing agent to reduce palladium acetate and obtain palladium nanoparticles. In the study conducted by Jia et al. [44], five different silanes were tested, namely PMHS, triethoxysilane ((EtO)_3_SiH), phenylsilane (PhSiH_3_), dietheylsilane (Et_2_SiH_2_) and dimethyl(phenyl)silane (PhMe^2^SiH). Except for (EtO)_3_SiH, the reported silanes were able to reduce the aldehydes into their respective alcohols. Similar to the aforementioned studies, silanes are known to be very active and most of the radicals are in contact with the gold chloride solution [45]. In a study of reduction of gold chloride on mesoporous silica, hydroxyl groups were identified as the reducing agent, gaining reducing capability via heating up to 80 °C [45]. Therefore, hydroxyl radical groups as well as free hydrogen radicals are believed to be responsible for the reduction of gold NPs. 

**Figure 2:**
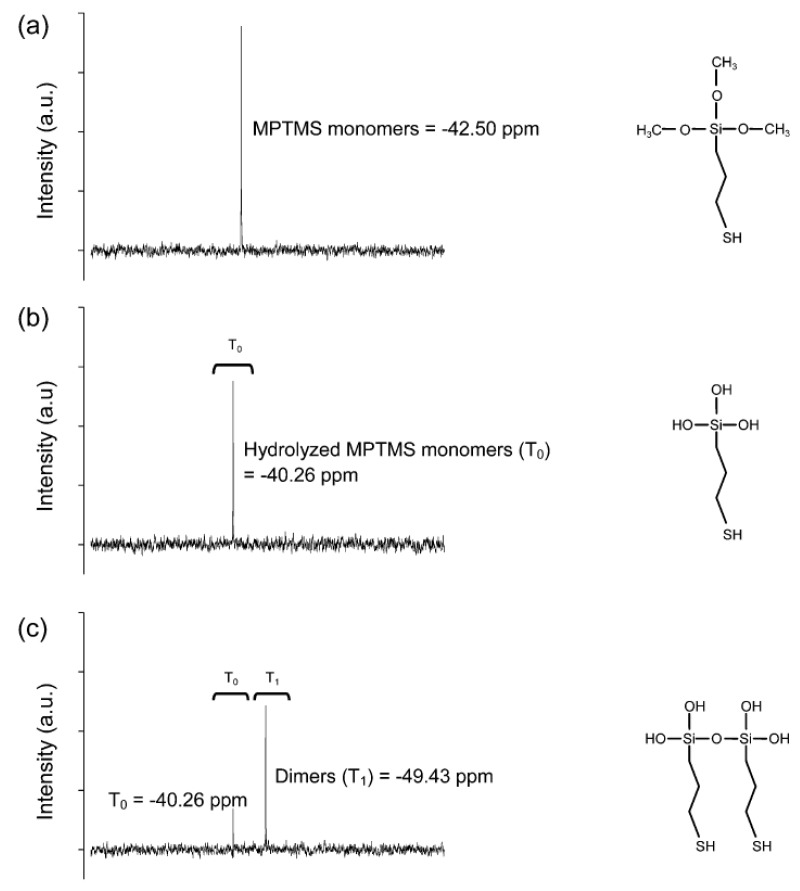
^29^Si nuclear magnetic resonance (NMR) spectra of (**a**) (3-Mercaptopropyl)trimethoxysilane (MPTMS) monomer, (**b**) hydrolyzed MPTMS monomer, (**c**) hydrolyzed MPTMS monomers and dimers.

The hydrolysis process is experimentally proven by the NMR results. ^29^Si NMR spectroscopy was employed to investigate the hydrolysis process of MPTMS as the precursor. As illustrated in Figure 2, hydroxyl (–OH) groups are equipped successfully after hydrolysis process, demonstrating the reducing property of hydrolyzed MPTMS. In the hydrolysis process, MPTMS was first added into water, and an oil–in–water mixture was formed by vortex. The mixed solution appeared turbid due to the presence of MPTMS droplets in water. Figure 2a shows the ^29^Si liquid NMR spectroscopy results for neat MPTMS (non-hydrolysed), which shows a single resonance peak at −42.5 ppm. During shaking for about 12 hours at room temperature, the droplets gradually disappeared to form clear solution. Figure 2b shows a new downshifted peak at T_0_ (−40.26 ppm peak), attributable to a strong presence of hydrolysed MPTMS. This is because with the three methoxy (–OCH_3_) groups (Figure 2a), MPTMS (HS(CH_2_)_3_Si(OCH_3_)_3_) is not soluble in water. MPTMS started to become water-soluble and form HS(CH_2_)_3_Si(OH)_3_ when hydrolyzing them to hydroxyl (–OH) groups (Figure 2b), as characterized by the ^29^Si NMR spectrum of the hydrolyzed MPTMS. After 12 hours of hydrolysis, Figure 2c shows a mixture of hydrolysed MPTMS monomers and dimers as evidenced by the presence of T_0_ and T_1_ (at −49.43 ppm) peaks, indicating a complete hydrolysis of MPTMS.

### 3.2. Characteristics of As-synthesized Au NPs

**Figure 3:**
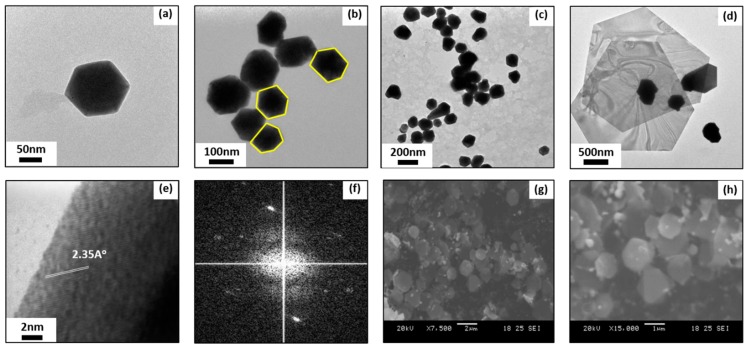
(**a**–**c**) Transmission electron microscopy (TEM) images of Au NPs under different magnifications, (**d**) high resolution TEM (HR-TEM) image, (**e**) selected area electron diffraction (SAED) pattern, (**f**) scanning electron microscope (SEM) image of Au Nanoplates.

Transmission electron microscopy (TEM) results (Figure 3a–c) show hexagonal Au NPs were successfully synthesized by the method. It has been found that Au NPs obtained by this method are hexagonal shaped. As-obtained Au NPs are six-sided polygonal shaped with an average size of 108 ± 11.3 nm. Based on high resolution TEM (HR-TEM) image of a single Au NPs and selected area electron diffraction (SAED) pattern, the spacing between clear lattice fringes (Figure 3d) is approximately 2.35A^o^. This spacing indicates that the synthesis of Au NPs commences preferentially at the (111) plane, corresponding to the works done by Joseph et al. [46] and Francis et al. [47] and suggests the formation of Au nanocrystal. Typically under 800 W power level of MW irradiation power, the mixture of 4 µl MPTMS and 1 mg HAuCl4 in 3 ml DI water could complete the reaction within 60 seconds and obtain hexagonal Au NPs. If the reaction is continued under MW irradiation after 60 seconds, ultimately two-dimensional hexagonal Au nanoplatelets could be obtained, as see in Figure 3d,g,h. Two-dimensional gold nanoplatelets are hexagonal shaped with the diameter of approximately 5 μm, while many small sized Au NPs are also found around these gold nanoplatelets. According to the aforementioned mechanism of Au NP formation and growth, it is believed that the formation of gold nanoplatelets, with the time of MW irradiation, is from Au NPs which continue absorbing residual AuCl_4_^−^ for further growth, after the nucleation of Au NPs.

**Figure 4:**
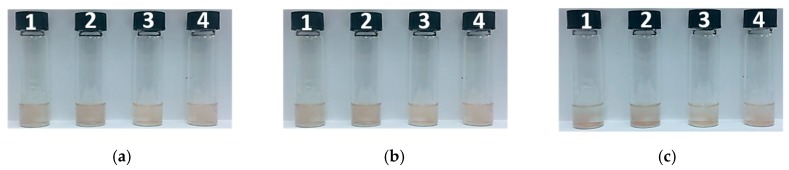
Stability test of as-synthesized Au NPs, (**a**) 1 hour, (**b**) 5 hours, (**c**) 10 hours. Solvent: 1 – DI water, 2 – methanol, 3 – ethanol, 4 – acetone.

The stability of as-synthesized Au NPs needs to be examined, as an important issue for further various applications. The purified Au NPs after washed by DI water were dispersed in 3 ml DI water, methanol, ethanol and acetone, respectively, with labels from 1 to 4. As seen in Figure 4, Au NPs in all solvent kept stable within the time of inspection, indicating as-synthesized Au NPs were very stable in these solvents.

**Figure 5:**
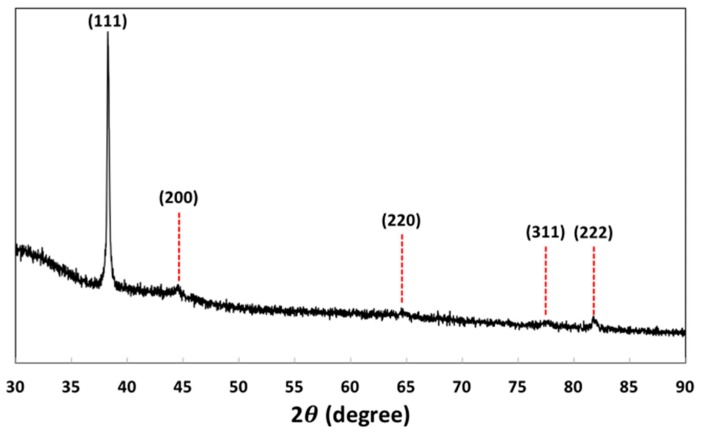
X-ray diffraction (XRD) X-ray Powder Diffraction pattern of Au NPs.

X-ray diffraction study (Figure 5) was conducted to acquire the crystalline nature of Au NPs. The XRD spectrum shows five diffraction peaks corresponding to the 2θ values of 38.25°, 44.58°, 64.55°, 77.69° and 81.76°, respectively. Such peaks can be assigned to (111), (200), (220), (311) and (222) reflections compared with JCPDS file No. 04-0784. It can be observed that the peak caused by (111) reflection is much more intense than those of (200), (220) and (311) reflections, clearly indicating and confirming that Au NPs are predominantly oriented along the (111) plane. With such highest peak diffraction at (111) plane, it can be also known that Au NPs formed by this method are pure crystalline nature.

### 3.3. Effect of Microwave Irradiation

**Figure 6:**
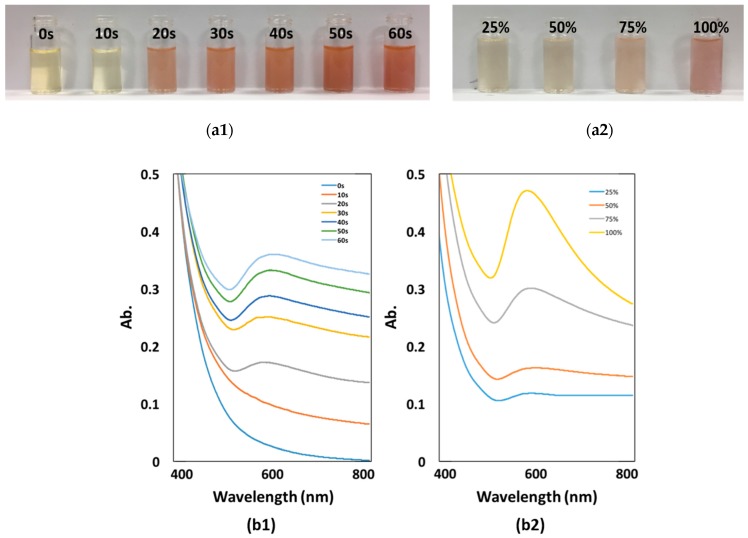
UV-vis spectra of Au NPs synthesized under (**a1**, **b1**) different MV MW irradiation time, (**a2**, **b2**) different MV irradiation power.

**Figure 7:**
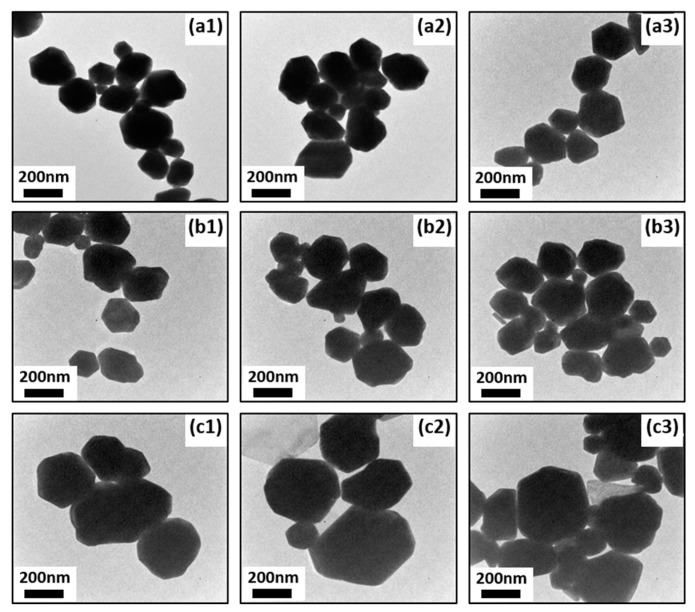
TEM images of Au NPs irradiated by various microwave (MW) MW irradiation times. (**a1**–**a3**) 20 s, (**b1**–**b3**) 40 s, (**c1**–**c3**) 60 s.

It is known that Au NPs manifest an intense absorption peak due to the surface plasmon resonance (SPR). Therefore, UV-vis absorption measurements were performed to evaluate the impacts of MV irradiation time and irradiation power on the synthesis of Au NPs. As illustrated in Figure 6a1, the color of solution becomes dark red from light yellow with the increase of irradiation time, indicating that more Au NPs were formed under longer MW irradiation. It is also seen in Figure 6b1 that the curves start to bulge from 20 second, which means the reaction started, consistent with the sample photo. In order to explore the impact of irradiation power on the reaction, solutions containing the same amount of HAuCl_4_ and hydrolyzed MPTMS were prepared and irradiated for 20 seconds under different irradiation power respectively. Figure 6a2,b2 show that the reactions under all irradiation power levels started very fast in an ultrafast time, but during the same time period higher irradiation power level leads to a larger amount formation of Au NPs. In Figure 7, it could be also seen that the averaged sizes of Au NPs increase with MV irradiation time, approximately from 200 nm after 20 seconds’ MW irradiation time to 500 nm after 60 seconds’ MW irradiation time, corresponding to the results from UV-vis spectra. 

### 3.4. Catalytic Reduction of 4-Nitrophenol

**Figure 8:**
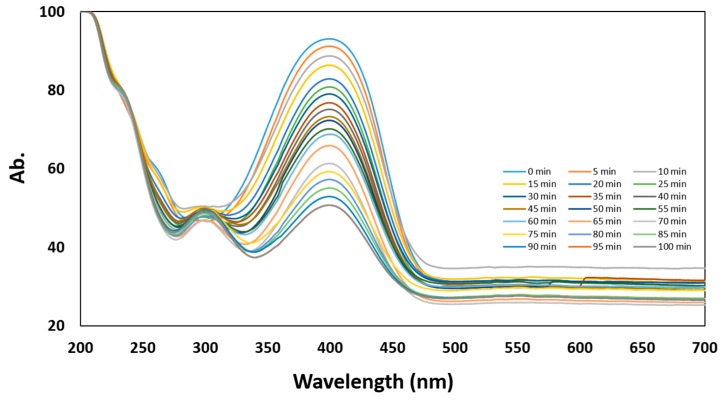
UV-vis spectra of reduction of 4-Nitrophenol using as-synthesized Au NPs.

In order to test the catalytic activity of as-synthesized Au NPs, the reduction of 4-nitrophenol was investigated. In the presence of NaBH_4_, there is a red shift of 4-NP from 317 nm to 400 nm, which is caused by the formation of 4-nitrophenolate ion in the alkaline medium due to NaBH_4_. Thus, the absorption peak at 400 nm was therefore monitored to track the reduction progress. In Figure 8, it was observed that after addition of Au NPs, the intensity absorption peak at 400 nm reduced significantly, while there was a new peak gradually appeared at 300 nm, indicating the conversion from 4-nitrophenol to 4-aminophenol. It is important to note that due to the low concentration of Au NPs added, the absorbance measurements of 4-NP are not affected by Au NPs. Moreover, the concentration of NaBH_4_ also exceeds greatly that of 4-NP; thus, it could be considered that the reduction rate can be independent of NaBH_4_ concentration. The reduction progress was monitored for 100 minutes and the test was conducted every 5 minutes. The reduction was observed to be progressing steadily due to the fading of the greenish-yellow color of the sample, corresponding to the work done by Gangula et al. [48]. Therefore, the as-synthesized Au NPs reduced by MPTMS are able to reduce 4-NP as an efficient catalyst and can be used further for various catalytic applications.

## 4. Conclusions

In this paper, a novel MW-assisted ultrafast and ultrafacile synthesis of Au NPs using hydrolysed organosilane MPTMS has been achieved in a very simple, rapid and energy-efficient route. For the first time, hydrolyzed MPTMS is utilized to synthesize hexagonal Au NPs under MW irradiation within seconds. The as-synthesized products include gold nanohexagons and two-dimensional hexagonal gold nanoplatelets, both of which are single-crystal with (1 1 1) planes as basal surfaces. The as-synthesized Au NPs exhibited a remarkable catalytic activity for the reduction of 4-nitrophenol, showing high potential to various catalytic applications. This environmentally friendly synthesis technique is potentially useful for fabricating unique metallic nanostructures in an ultrafast period of time, promising for large scale production.
